# Utilization of Optical OFDM Modulation on Blue LED VLC Datacom Without Equalization for 4 m Wireless Link

**DOI:** 10.3390/mi15111322

**Published:** 2024-10-30

**Authors:** Yuan-Zeng Lin, Chien-Hung Yeh, Wen-Piao Lin, Chi-Wai Chow

**Affiliations:** 1 Department of Photonics, National Yang Ming Chiao Tung University, Hsinchu 300093, Taiwan; 2 Department of Photonics, Feng Chia University, Taichung 407802, Taiwan; 3 Master’s Program of Space System Engineering, Feng Chia University, Taichung 407802, Taiwan; 4 Department of Electrical Engineering, Chang Gung University, Taoyuan 333323, Taiwan; wplin@mail.cgu.edu.tw

**Keywords:** visible light communication (VLC), OFDM-QAM, blue LED, APD, equalization

## Abstract

To achieve higher visible light communication (VLC) traffic capacity, using the wide bandwidth light-emitting diode (LED) and spectral efficiency modulation signal, is currently the most commonly used method. In this demonstration, we apply the orthogonal frequency division multiplexing quadrature amplitude modulation (OFDM-QAM) with bit- and power-loading algorithm on single blue LED to achieve >1 Gbit/s VLC capacity, when a 400 MHz bandwidth avalanche photodiode (APD)-based receiver (Rx) is exploited for decoding. Here, the higher sensitivity APD can be applied to compensate for the wireless VLC link length in the proposed LED VLC system, and due to the lower LED illumination (255 to 40 lux), is used for the indoor access network after passing the wireless link length of 1 to 4 m. As a result, using single blue LED can achieve 0.962 to 1.057 Gbit/s OFDM rate with available 400 MHz bandwidth APD in poorly illuminated condition indoors without applying analogy equalization.

## 1. Introduction

Recently, the universal applications of 5G and optical access in many industries and the deep integration of ICT and AI revolutions will spur the whole of humanity to regularly cause the generation of informatization, digitalization and intelligence. The demand of broadband data capacity, which is one of the main performance indicators, will continue to grow beyond the fifth-generation/sixth-generation (B5G/6G) mobile access methods because of many data consumption applications, such as augmented/virtual reality (AR/VR), automatic driving and cloud computing [1,2]. Furthermore, to increase and achieve the convenience and connection of a broadband B5G/6G network, the indoor visible light communication (VLC) transmission technology will be also a good candidate [3,4]. The VLC technology based on a light emitting diode (LED) device is emerging know-how to facilitate high speed and wide capacity access connectivity [5,6,7]. In recent years, VLC-related technologies have developed rapidly in basic research and commercial applications. Therefore, the Japan-based Visible Light Communication Consortium (VLCC) was created in 2002 to promote VLC [8]. The Center for Ubiquitous Communication by Light (UC-Light) sponsored by the University of California (UC) system has been started in the United States [9]. It is to permit wireless communications by adding signals into the light by the LED devices for traffic control, illumination, display backlight, advertising and other objects. The Smart Lighting Engineering Research Center has been recognized to expand new skills of the optical wireless link to the internet connection with energy-efficient function [10]. The OMEGA Project, supported by the European Commission at the Seventh Research Framework Programme (FP7), was initiated to develop the VLC system in 2008 [11]. IEEE 802.15.7 standard: Short-Range Wireless Optical Communication Using Visible Light) was completed in 2011 to enhance market require and commercialize the VLC development [12]. This standard concluded the physical layer (PHY) air interface and the medium access control (MAC). In addition, from 2018 until now, the development of VLC-related technology has been completed according to the formulation of the IEEE 802.11bb standard for Light Fidelity (LiFi) [13]. LiFi is an optical wireless link that utilizes lightwave rather than radio frequencies to broadcast data signals. By connecting the light spectrum, LiFi can release fast and reliable optical wireless communication with unmatched security compared to traditional wireless technologies of WiFi, 5G and B5G.

As we know, the improvements in power efficiency and cost reductions of the LEDs have expanded the use of LEDs to many application areas, such as display backlights, automobile lights, traffic lights and both indoor and outdoor lighting. The LEDs have the gains of power efficiency, compact size, cost-effectiveness, long lifetime and easy to be integrated in different products. Therefore, as more and more LEDs are used to implement the main function of lighting, the additional communication functions can be applied at little additional cost. The VLC technology has increased substantial concentration owing to its many benefits over out-of-date communication ways, such as radiofrequency (RF) communication. The VLC-induced benefits involve an enlarged bandwidth, improved security and the ability to operate in spaces free from electromagnetic interference. However, the available modulation bandwidth of phosphor-based LEDs will limit the useful data rate for signal access [14,15]. The phosphor-based LED VLC could limit the traffic rate from a few Mbit/s to hundreds of Mbit/s [16,17]. Therefore, to enhance the LED-based VLC data capacity, utilizing the spectral efficiency modulations [18,19], higher modulation bandwidth LED [20,21], wavelength-multiplexing-division (WDM) red-green-blue (RGB) LEDs [22,23], polarization-multiplexing LED [24,25] and parallel VLC transmission system [26] have been investigated and discussed experimentally. In addition, the VLC signal connection must be able to obtain a better optical signal-to-noise ratio (SNR) under sufficient illumination for the high traffic rate.

In 2007, Park et al. presented a 16 × 16 red and green array LEDs with 32 drivers to deliver 4 Mbit/s and 10 Mbit/s on–off keying (OOK) data rates with detected power of >−8.5 dBm based on a visible light signboard under the corresponding free space link length of 4.2 and 3.6 m, respectively [27]. However, they needed higher illumination to detect and decode the VLC signal. In 2008, Le-Minh et al. demonstrated a 40 Mbit/s OOK VLC data rate by applying 16 phosphor LEDs with pre-equalization at multiple-resonant modulation in the VLC System when the 3-dB VLC bandwidth was extended to 25 MHz [28]. In 2008, the same group of Le-Minh et al. improved the pre-equalization technology and included a blue filter at receiver (Rx) site to increase the white LED modulation bandwidth to 45 MHz for 80 Mbit/s OOK VLC rate with low bit error rate (BER) [15]. In 2009, Le-Minh et al. also presented a 100 Mbit/s OOK VLC connection by optimizing the electrical circuits of pre- and post-equalizations to achieve a 50 MHz effective modulation of single white LED [17]. The LED VLC bandwidth enhancement technology proposed above was caused by using analogy equalization circuit design and adding a blue light filter before entering the Rx. In addition, these methods only increased the LED VLC modulation capacity by about two times.

In 2011, Chow et al. demonstrated a 10 Mbit/s OOK-NRZ VLC transmission system by using a simple first-order resistance–capacitance equalization circuit based on a 1 MHz bandwidth phosphor LED component and pre-distorted OOK signal modulation format [16]. This demonstrated white LED VLC system could enhance 10 times modulation rate. To increase the LED VLC modulation rate to 20 times, the same group designed the quaternary-amplitude-shift-keying (4-ASK) modulation with digital filtering to enhance the direct modulation speed of VLC system [29]. Thus, the proposed SRRC filter achieved a spectral efficiency of 2 bit/Hz, and then, the 4-ASK VLC signal attained a spectral efficiency of 2 bit/Hz. The total spectral efficiency was about 4, and the 20 Mbit/s traffic rate was transmitted within a 5 MHz bandwidth. The two demonstrated LED VLC technologies did not apply the blue filter in the Rx site to enhance the modulation bandwidth. In 2013, Yeh et al. first demonstrated real-time phosphor white LED VLC connection with 37 Mbit/s signal throughput after a 1.5 m wireless transmission distance [30]. They integrated an orthogonal frequency division multiplexing (OFDM)-based digital signal processing (DSP) chip and analog front end (AFE) for real-time VLC link, while the modulation bandwidth was extended from 1 to 12 MHz without using blue filter. The above also illustrated the practicality and high applicability of the LED VLC transmission system.

Then, in order to obtain a higher LED VLC modulation rate, some related studies used the hybrid red, green, and blue (RGB) LEDs used for wavelength-division-multiplexing (WDM) investigation [23,31,32]. Since there is no bandwidth narrowing caused by the phosphor effect, the usable modulation bandwidth of RGB LEDs will be better than that of phosphor LEDs. In 2017, Zhang et al. proposed RGB LEDs to achieve total rate of 4.05 Gbit/s by applying PS-Manchester coded Nyquist PAM-8 modulation format, when the bandwidth of GRB LEDs of 960, 830 and 910 MHz were achieved [33]. In addition to RGB-LED VLC systems, single R, G or B LEDs are also often used in research area for VLC experiments and analysis. Moreover, using micro-LEDs to achieve modulation rates of approximately Gbit/s is also a new trend in research in recent years, such as G-LED or B-LED [34,35]. As long as the available modulation bandwidth of MLED can reach approximately hundreds of MHz to GHz. Furthermore, according to the previous studies of blue LED VLC systems [36,37,38], the achievable modulation bandwidth of LED was between 225.4 and 991 MHz to reach the traffics of 660 Mbit/s to 2 Gbit/s over the corresponding VLC transmission distance of 0.5 [38] to 2.3 m [36]. This means that the high and sufficient illumination was required to increase the data rate and transmission length of VLC signal. Thus, how to deliver higher LED VLC data under lower illuminance will be an important issue to be overcome.

In this demonstration, we apply a blue LED serving as VLC transmission signal indoors to reach data rate of >Gbps by using the orthogonal frequency division multiplexing quadrature amplitude modulation (OFDM-QAM) with bit- and power-loading design and 400 MHz bandwidth avalanche photodiode (APD) for decoding signal. To focus and enhance the detected illumination, we utilize a focusing lens in front of LED to reduce the divergency diameter of VLC signal on the receiver (Rx) site. Therefore, over the available 400 MHz bandwidth of APD, the VLC modulation rate of 0.962 to 1.057 Gbit/s cab be attained under the corresponding illumination of 40 to 255 lux without using analog equalization on the transmitter (Tx) and Rx site. Moreover, the wireless VLC traffic length of 1 to 4 m is reached based on the proposed blue LED VLC architecture in the case of insufficient indoor light.

## 2. Experiment and Results

**Figure 1:**
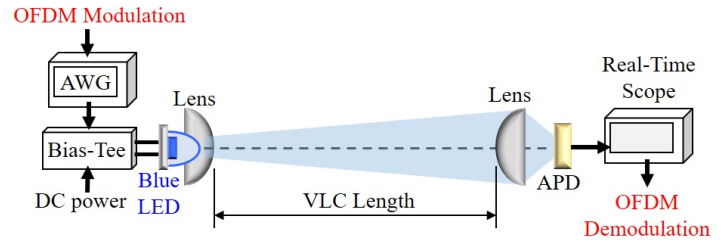
Experimental setup of the presented blue LED VLC transmission system.

Figure 1 presents the experimental setup of VLC transmission system by utilizing the blue LED and 400 MHz bandwidth silicon (Si) avalanche photodiode (APD, *Thorlabs*) as optical Tx and Rx, respectively. To carry the OFDM-QAM modulation signal on the blue LED [24], a proper bias-tee (BT) with 3 GHz operation bandwidth is applied to connect to the DC current and AC signal for lighting and signaling. In the measurement, the bias current of blue LED is set at 70 mA. As shown in Figure 1, a focusing lens with diameter of 50 mm on the Tx side can result in a narrow divergence diameter on the Rx site for increase the detected optical signal to noise ratio (SNR) of VLC traffic. Besides, to focus and enhance the detected LED VLC illumination through the 400 MHz APD, a focusing lens also with diameter of 50 mm is placed in front of the APD, respectively, so that the light power emitted by the LED can be collected more concentratedly. In the measurement, an arbitrary waveform generator (AWG) with analog bandwidth of 20 GHz and sample rate of 50 GS/s (*Tektronix*) is employed to cause and generate the OFDM-QAM modulation signal through the Matlab programming. The produced OFDM-QAM also has bit- and power-loading design to operate on the blue LED for delivering VLC. To obtain the best output signal-to-noise ratio (SNR) and bit load output, the adaptive bit- and power-loading algorithm is applied for coding and decoding. Here, the cyclic prefix (CP), data length, training length, fast Fourier transform (FFT) size and OFDM subcarrier number used are 32, 500, 50, 512 and 90 in the experiment, respectively. Through a length of wireless VLC transmission, the OFDM VLC signal is received by an APD directly to transfer to electrical signal and decoded by using a real-time scope with analog bandwidth of 16 GHz and sample rate of 80 GS/s (*Teledyne Lecroy WaveMaster*). Therefore, the received electrical OFDM signal is also captured by applying offline Matlab programs for the decoding signal. To simplify and reduce the complexity of the proposed VLC transmission system, we do not use any equalization and filtering technologies on the optical Tx and Rx modules to demodulate the signals.

**Figure 2:**
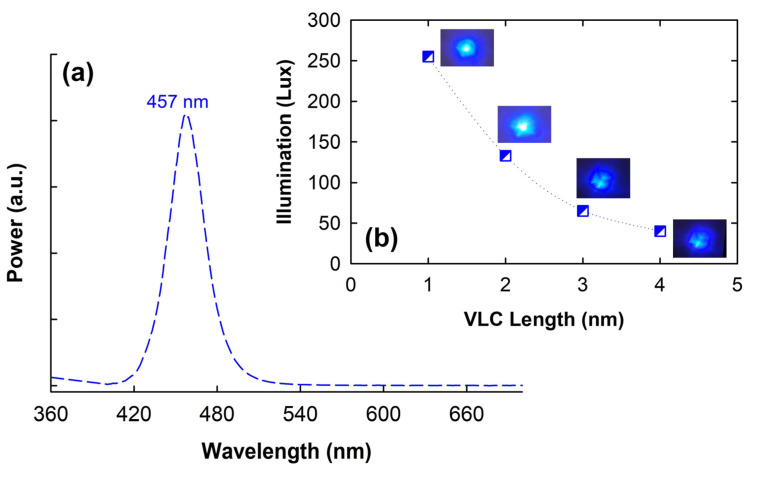
(**a**) Measured output spectrum of blue LED. (**b**) Detected illumination of LED after passing through 1 to 4 m free space link length, respectively. Insets are the photos of corresponding light spots through different VLC link lengths.

First, Figure 2a displays the measured output spectrum of blue LED. The center wavelength of the LED is around 457 nm. To realize the obtainable illumination of the LED indoors, a power meter (PM) is employed for measurement after an optical wireless transmission length of 1 to 4 m without going through the lens of the Rx site, respectively. Figure 2b shows the observed illumination of blue LED through various wireless VLC link lengths, and the detected illumination of the blue LED is 255, 133, 64 and 40 lux through the VLC link of 1, 2, 3 and 4 m, respectively. Furthermore, the insets of Figure 2b are the corresponding photos of light spots under various free space link lengths. Due to the use of lenses with a smaller divergence angle, the observed spot diameter would be less than 8 cm after a 4 m VLC link length, as seen in the insets of Figure 2b. In addition, the corresponding spot diameters of 5.5, 6, 6.5 and 8 cm are observed after through a free space link length of 1, 2, 3 and 4 m, respectively. As we know, the valuable illumination would influence the optical signal-to-noise ratio (SNR) of the detected OFDM signal. The obtained results of Figure 2b will help to understand the verification of the next VLC transmission experiment.

**Figure 3:**
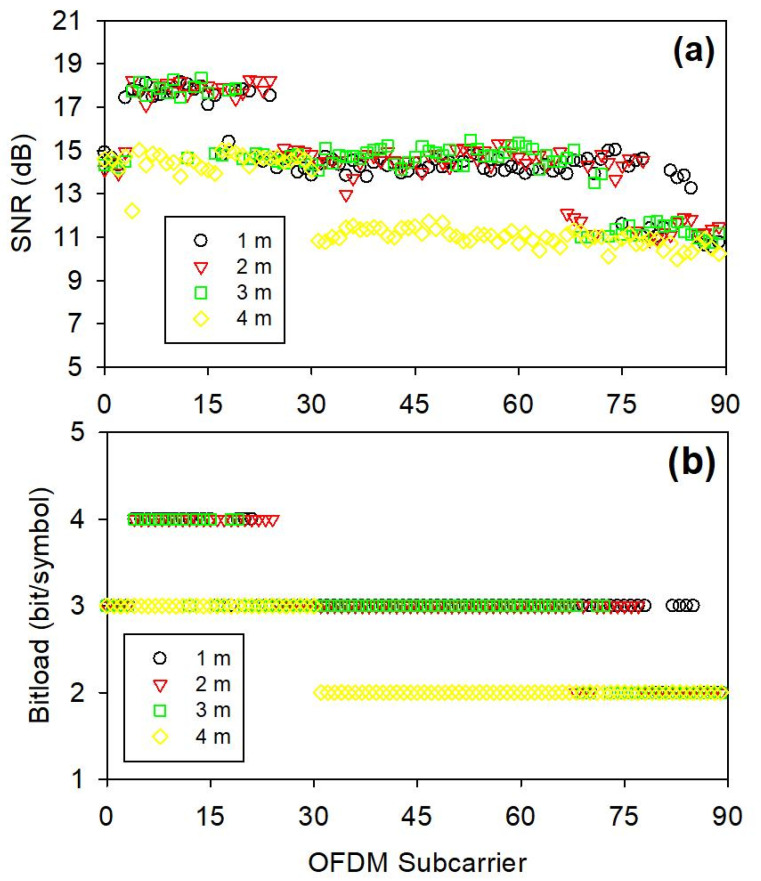
(**a**) Corresponding SNR and (**b**) bit/symbol of each OFDM subcarrier measured over a 400 MHz bandwidth after 1 to 4 m free space VLC transmission lengths, respectively.

Then, to execute and generate the OFDM signal in the presented blue LED VLC system, the fast Fourier-transform (FFT) size, subcarrier number and cyclic prefix (CP) is set at 512, 90 and 32 over the effective bandwidth of 400 MHz, respectively. To understand the SNR performing of each OFDM subcarrier, Figure 3a exhibits the observed results after transmitting through different free space connection lengths within the available 400 MHz bandwidth. In the measurement, the SNRs of subcarriers are achieved between 10.54 and 18.15 dB, 10.86 and 18.27 dB, 10.73 and 18.35 dB and 10.24 and 15.01 dB through 1, 2, 3 and 4 m wireless VLC links, respectively, while the optimal and adaptive bit and power loading algorithm is applied. Here, the detected illumination of LED VLC signal can be enhanced after traveling through the focusing lens on the Rx site. Since the Si APD only has an available modulation bandwidth of 400 MHz, the SNR frequency range measured in Figure 3a is the obtainable bandwidth of the blue LED in this measurement. Figure 3a shows that all the measured SNR of OFDM carrier is greater than 10.24 dB within 400 MHz frequency bandwidth after through 4 m free space link. The experimentally measured SNR range will decrease as the VLC transmission distance increases. This is because the detected optical power will also decrease accordingly. Next, according to the obtained SNRs in the presented blue LED VLC transmissions, the corresponding bit number per symbol of 1 to 4 is also achieved, respectively, as illustrated in Figure 3b. Only under a wireless transmission distance of 4 m, the resulting bit number is distributed between 1 and 3. Since a higher SNR can result in a better bit number. Therefore, under a shorter VLC transmission distance, the overall bit number can be higher, as also seen in Figure 3b.

**Figure 4:**
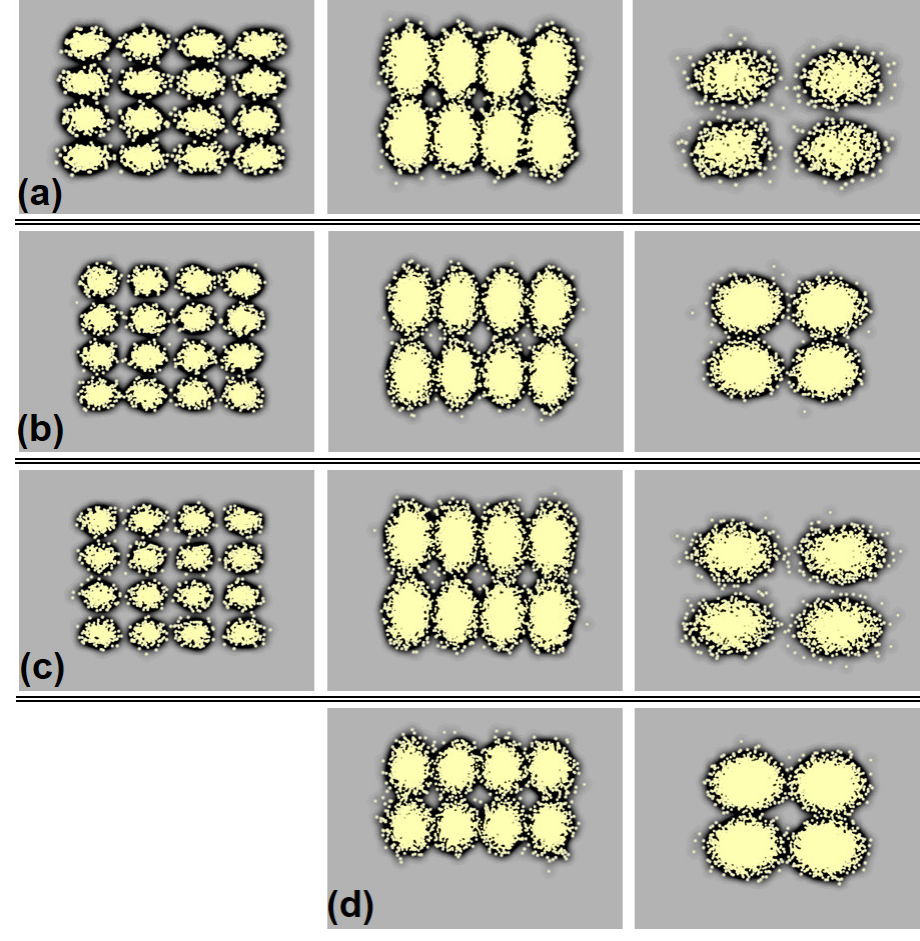
The measured constellation performance of OFDM-QAM VLC signal after passing through (**a**) 1, (**b**) 2, (**c**) 3 and (**d**) 4 m free space link length, respectively.

In the demonstration, the corresponding constellations of VLC OFDM-QAM signal under 4- to 16-QAM are also exhibited in Figure 4a–d after 1 to 4 m wireless VLC link lengths, respectively. The constellation measured is based on the obtained SNR of the corresponding OFDM signal after passing through different wireless link length. Figure 4a–d presents the observed constellation distributions of OFDM signals are clear after even after a 4 m length of VLC connection.

**Figure 5:**
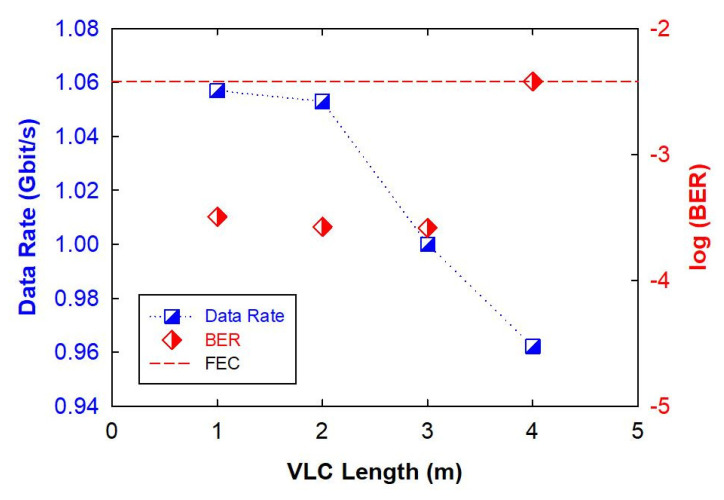
Measured VLC traffic rate and BER under the VLC link length of 1 to 4 m, respectively.

According to the obtained results of Figure 3, the achievable OFDM VLC data rates of 1.057, 1.053, 1 and 0.962 Gbit/s can be reached through 1, 2, 3 and 4 m free space connection lengths under the matching illuminations of 255, 133, 64 and 40 lux, respectively, as seen in Figure 5. Moreover, the detected bit error rates (BERs) of these VLC traffic rates can be also less than 3.8 × 10^−3^ to meet with the forward error correction (FEC) target, as displayed in Figure 5. The corresponding results show that the VLC data rate can be greater than 0.962 Gbit/s via utilizing single blue LED device, when the detected illumination is 40 lux after 4 m wireless VLC link. As seen in Figure 5, as the transmission distance of VLC becomes longer, the available traffic capacity will also become smaller due to the insufficient illumination (SNR). Consequently, the presented blue LED VLC system can achieve 0.962 to 1.057 Gbit/s data rate by utilizing the OFDM-QAM modulation with bit- and power-loading algorithm and 400 MHz bandwidth APD without analog equalization circuit on the Tx and Rx sites. Figure 2b is the measurement of the illumination of the most central position of the LED at a wireless transmission distance of 1 to 4 m. We know that, under the proposed VLC system, as long as the VLC light is collected through the focusing lens of the Rx site. Therefore, this also means that even under different transmission distances, as long as the illumination received is above 40 lux, the OFDM capacity can reach 0.962 Gbit/s. In this demonstration, there is no requirement for VLC signal alignment and transmission. We only need to consider the detected LED illumination for VLC connection.

To achieve higher modulation rate of blue LED VLC, past studies [38,39,40] required wide modulation bandwidth, higher VLC illumination and modulation format with special coding. They required an effective modulation bandwidth greater than 500 MHz. Compared to similar literature in the past [38,39,40], the proposed blue LED VLC system can achieve a 0.962 Gbit/s OFDM data rate within a modulation bandwidth of 400 MHz under a weak illumination of 40 lux after a transmission distance of 4 m. Moreover, the presented VLC system does not need to add extra equalization on the Tx and Rx side and apply special modulation technology. Thus, we believe that the proposed LED VLC system not only has a simple transmission architecture but also has a cost-effective feature.

## 3. Conclusions

In summary, we proposed and demonstrated a VLC transmission system indoors based on single blue LED and 400 MHz bandwidth APD. To achieve the spectral efficiency operation in the presented VLC system, we applied the OFDM-QAM modulation format with bit- and power-loading design on the blue LED to achieve high data capacity. To increase the obtained SNR of VLC signal, a focusing lens was placed in front of LED to narrow the light spot diameter for enhancing the received illumination. Hence, the OFDM VLC traffic rate of 0.962 to 1.057 Gbit/s was reached by using single blue LED within 400 MHz bandwidth after transmitting through 1 to 4 m free space connection length. In addition, the detected illumination of 40 to 255 lux corresponded to the wireless VLC length of 1 to 4 m. As a result, according to the demonstrated blue LED VLC system, the smallest data rate of 0.962 Gbit/s was obtained at the very low illumination of 40 lux through 4 m optical wireless connection length for indoor signal access.

## Data Availability

The data that support the findings of this study are available from the corresponding author upon reasonable request.
